# Founding weaver ant queens (*Oecophylla longinoda*) increase production and nanitic worker size when adopting non-nestmate pupae

**DOI:** 10.1186/2193-1801-4-6

**Published:** 2015-01-06

**Authors:** Issa Ouagoussounon, Joachim Offenberg, Antonio Sinzogan, Appolinaire Adandonon, Dansou Kossou, Jean-François Vayssières

**Affiliations:** 4 Faculté des Sciences Agronomiques, Université d’Abomey Calavi, 03 BP 2819 Jéricho Cotonou, Kragujevac, République du Bénin; 5 Department of Bioscience, Aarhus University, Vejlbovej 25, 8600 Silkeborg, Denmark; 6 Ecole Nationale Supérieure des Sciences et Techniques Agronomiques (ENSTA) de Kétou, Université d’Agriculture de Kétou, BP 95 Kétou, Kragujevac, République du Bénin; 7 CIRAD, UPR HortSys, 34398 Montpellier, France; 8 IITA, Biocontrol Unit for Africa, 08 BP 0932 Cotonou, Kragujevac, République du Bénin

**Keywords:** Biological control, Colony growth, Entomophagy, Nanitic worker size, *Oecophylla longinoda*, Weaver ant farming

## Abstract

Weaver ants (*Oecophylla longinoda* Latreille) are used commercially to control pest insects and for protein production. In this respect fast colony growth is desirable for managed colonies. Transplantation of non-nestmate pupae to incipient colonies has been shown to boost colony growth. Our objectives were to find the maximum number of pupae a founding queen can handle, and to measure the associated colony growth. Secondly, we tested if transplantation of pupae led to production of larger nanitic workers (defined as unusually small worker ants produced by founding queens in their first batch of offspring). Forty-five fertilized queens were divided into three treatments: 0 (control), 100 or 300 non-nestmate pupae transplanted to each colony. Pupae transplantation resulted in highly increased growth rates, as pupae were readily adopted by the queens and showed high proportions of surviving (mean = 76%). However, survival was significantly higher when 100 pupae were transplanted compared to transplantation of 300 pupae, indicating that queens were unable to handle 300 pupae adequately and that pupae require some amount of nursing. Nevertheless, within the 60-day experiment the transplantation of 300 pupae increased total colony size more than 10-fold whereas 100 pupae increased the size 5.6 fold, compared to control. This increase was due not only to the individuals added in the form of pupae but also to an increased per capita brood production by the resident queen, triggered by the adopted pupae. The size of hatching pupae produced by the resident queen also increased with the number of pupae transplanted, leading to larger nanitic workers in colonies adopting pupae. In conclusion, pupae transplantation may be used to produce larger colonies with larger worker ants and may thus reduce the time to produce weaver ant colonies for commercial purposes. This in turn may facilitate the implementation of the use of weaver ants.

## Introduction

Weaver ants (*Oecophylla* spp.) are utilized for biological control of insect pests in a number of tropical tree crops (Peng et al. 2004, 2010; Van Mele et al. 2007) and are in this way known to improve fruit quality (Sinzogan et al. 2008; Peng et al. 2004). They are therefore increasingly being utilized as a substitute for synthetic chemical pesticides (Dwomoh et al. 2009; Offenberg et al. 2013). Furthermore, *Oecophylla* ants are used as human food and as feed for animals (Césard 2004; Sribandit et al. 2008; Offenberg 2011; Van Huis et al. 2013). For each of these uses, the availability of mature ant colonies is essential. *Oecophylla* ant-colony rearing under ambient tropical conditions takes 2–3 years before a young colony becomes mature and ready for commercial use (Vanderplank 1960; Peng et al. 1998; Peng et al. 2013; Offenberg and Wiwatwitaya 2010). However, this period can be shortened, if early colony growth can be boosted.

Ants employ two different ways to increase early colony growth, firstly, *Oecophylla* and other ant species are known to found new colonies with multiple queens (pleometrosis) in order to increase the probability of survival during the initial phase of colony development via a faster production of more workers (Peeters and Andersen 1989; Peng et al. 1998; Bernasconi and Strassmann 1999; Offenberg et al. 2012a). Secondly, adoption of non-nestmate brood from other colonies may increase colony growth as several ant species are known to rob intraspecific brood from neighbouring colonies and in this way accelerate colony growth by adding these robbed individuals to their worker force (Bartz and Hölldobler 1982; Rissing and Pollock 1987). Recent studies have shown that artificial transplantation of pupae to young weaver ant colonies can be utilized to boost their growth (Krag et al. 2010; Offenberg et al. 2012b; Peng et al. 2013; Ouagoussounon et al. 2013). However, the maximum number of pupae that can be nursed by, and therefore transplanted to, a sole founding queen is not yet known.

Not only colony size but also worker size variation is an important component of labor division in colonies of *Oecophylla* ants (Dejean 1991). The worker force includes two castes, minor and major workers. Small minor workers are most important in rearing brood, whereas large workers are more important in nest construction, territory defense and foraging for insect prey and transport of sexual brood (Crozier et al. 2010; Dejean 1991), the latter tasks becoming more important with colony size. Brian (1957) and Wood and Tschinkel (1981) indicated colony size as a major factor affecting worker size. Brian (1957) reported that worker size gradually increases in the monomorphic *Myrmica rubra* until the colony reached about 300 workers, or 10% of its mature size. Wood and Tschinkel (1981) described the appearance of worker size variation in colonies of *Solenopsis invicta* of up to about 6000 workers showing that worker head width increase gradually as the colony grows. Rissing (1987) reported that the mean worker size increases during the first year in the polymorphic *Veromessor pergandei*, but he did not record colony size. Peng et al. (2004) showed that small *Oecophylla smaragdina* colonies less than 1.5 years old produce smaller and slimmer workers (nanitics). As colony task repertoire and thus the benefits derived from *Oecophylla* colonies may increase with worker size range and as worker size (and thus size range) may increase with increasing number of workers in a colony, the transplantation of pupae may lead to better performing colonies. The hypothesis that transplantation of pupae leads to production of larger workers should therefore be tested.

In this study we tested: (i) the effect of pupae transplantation on queen brood production, (ii) whether pupae survival was affected by the transplantation of high numbers of pupae (300 pupae per queen), and (iii) whether pupae transplantation led to the production of larger intrinsic workers.

## Materials and methods

### Biological material and general experimental design

In a mango plantation in the Parakou area (09° 37' 01"N/02° 67' 08"E) of Benin 45 *Oecophylla longinoda* queens were collected after their nuptial flight with the use of artificial nests made of rolled leaves (Ouagoussounon et al. 2013). Queens were collected from mango trees 2–3 times a week during the mating season. Thus, all queens were collected 1–3 days after their mating flight. At this developmental stage all colonies were composed of a single queen and her eggs. After collection, the queens and eggs inside the rolled leaves were put into small transparent PVC plastic containers (diameter = 4.5 cm; height = 10.5 cm) sealed with mesh nylon net at the open end. After some time leaves dried up and the dry leaves were removed. A second leaf was provided when queens were transferred to the larger container (see below). After that no additional leaves were provided as the humidity the leaves provided was only needed during the initial phase of colony founding. The forty-five mated queens were divided into three pupae transplantation treatments with 0, 100 or 300 non-nestmate pupae being transplanted to each queen, respectively, and resulting in 15 replicates per treatment. Every time a new queen was collected in the field, it was sequentially allocated to one of the three treatments. The colonies were separated by placing each of them in a container (see below) on an “island” composed of a concrete block (10×10×15 cm) placed in a tray with water. The spacing between islands was 25 cm × 20 cm. All colonies were kept at ambient temperature ranging between 24.6°C and 30.9°C (mean = 27.8°C ± 2.68 SD) on a table. As a secondary protection against intruding ants each table leg was placed in a tray with water. During the experiment, all colonies were provided with a few drops of pure water every day to allow the queens to drink. After the emergence of the first imago workers, 20% sucrose water was provided to each colony *ad libitum* every day. One week after the emergence of imago workers, protein food in the form of canned fish and insects was provided in similar amounts and proportions to all colonies *ad libitum*; newly eclosed workers usually only show limited interest in protein during the first week. The number of eggs laid by each queen until pupae were transplanted was recorded and used as a covariate in the statistical analyses described below.

### Pupae adoption

Pupae transplantation took place when egg-laying stopped (approximately 5–7 days after the nuptial flight). Non-nestmate pupae were obtained from several mature *O. longinoda* colonies by cutting down ant nests, breaking them up and gently sucking up pupae with an aspirator. Pupae were kept in plastic containers on a table under ambient conditions until transplanted into the experimental colonies. All pupae were transplanted on the same day as they were collected to ensure equal pupae quality. Only pupae of major workers were transplanted. Pupae containing major workers were distinguished from minors by the size of the pupae. During transplantation, each colony (including the control colonies) inside the rolled leaf was transferred from the small container to a cylindrical large transparent PVC plastic container (diameter = 8 cm and height = 5 cm) with a mango leaf and the relevant number of pupae placed inside the container. The queens’ behavior toward transplanted pupae was observed after pupae transplantation. Based on the number of live adopted imago workers present after 60 days (see below), the proportions surviving from the total number of transplanted pupae (100 or 300 pupae) into imago workers was calculated and arcsine transformed pupae survival were compared between treatments with ANOVAs. As the total number of added pupae was used for the survival analysis, mortality included those pupae that were discarded by the queens in the treatment with 300 pupae (see Results). An additional analysis was conducted on the survival of only those pupae that was kept by the queens.

### Brood production

The large containers allowed inspection and counting of brood in their different developmental stages with the aid of a magnifying glass. The numbers of intrinsic eggs, larvae, pupae and imago workers (defined as the brood and workers produced by the resident queens) as well as adopted workers, were estimated non-destructively 60 days after the pupae transplantation in all the colonies. Exact counts of brood were possible in treatments with 0 and 100 pupae. In the treatment with 300 pupae brood numbers were approximated due to the difficulty of assessing exact numbers in the dense piles of brood. At this point all adopted pupae and the oldest intrinsic brood had developed into imago workers. However, intrinsic imagines could be distinguished from adopted imagines due to the size difference between the transplanted workers from the mature colony and the much smaller nanitic workers produced by the founding queens (Porter and Tschinkel 1986; Peng et al. 2004). For comparison, head width and length of transplanted major pupae were 1.45-1.5 mm and 8–9 mm, respectively. Mean numbers of brood individuals were compared between treatments with ANOVAs.

### Worker size

To investigate the effect of transplantation on nanitic worker sizes, four colonies were randomly selected in each pupae transplantation treatment, leading to 12 colonies in total. Pupae length (from the head to the gaster) and head width (across the eyes) of the intrinsic hatching pupae were measured on 10 individuals from each of the four colonies in each treatment. Worker size data were log_10_(x) transformed which produced normal distributions and variance homogeneity before testing differences between treatments with ANOVAs. As workers originating from the same colonies may not be considered independent, an additional analysis based on colony average worker size was performed. JMP 10.0.0 statistical software was used for all statistical analyses.

## Results

### Pupae adoption

In the colonies that received foreign pupae, queens moved out of their rolled leaf nests after the transplantation and placed the transplanted pupae in a pile which they subsequently guarded and nursed. However, in the colonies receiving 300 pupae, queens removed and threw out some pupae from the plastic container. No pupae were removed by the queens in the 100 pupae treatment whereas between 17 and 45 pupae were removed by the queens in the 300 pupae treatment (mean ± SD = 32.1 ± 8.7). As these pupae drowned in the surrounding water their potential survival could not be determined. Survival from transplanted pupae into imago workers ranged between 63% and 91% (mean % survival ± SD = 76.2 ± 8.32). Survival, though, was significantly lower when 300 pupae were transplanted (mean % survival ± SD, 100 pupae = 83.5 ± 3.89, 300 pupae = 68.9 ± 3.95; ANOVA including egg numbers before transplantation as a co-factor, F_(1, 27)_ = 94.3; *P* < 0.0001). There was also significantly lower survival at the 300 pupae transplantation rate if only the survival of the pupae that were kept by the queens were considered (mean % survival of pupae ± SD, 100 pupae = 83.5 ± 3.89, 300 pupae = 77.2 ± 3.92; ANOVA including egg numbers before transplantation as a co-factor, F_(1, 27)_ = 19.18; *P =* 0.0002).

### Brood production

**Figure 1:**
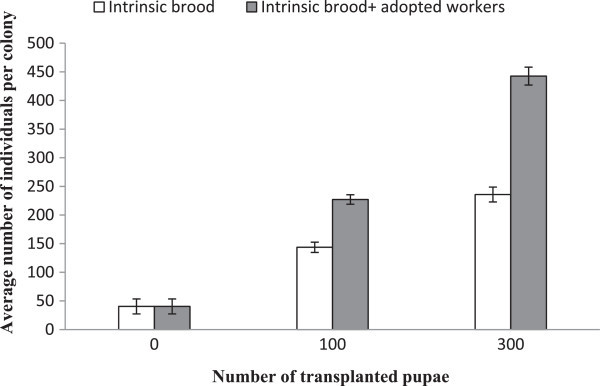
**The mean (± SD) number of individuals (eggs, larvae, pupae and imago workers) per colony, 60 days after the transplantation of pupae.**

Transplantation of pupae led to a significant increase in the per capita production. This was true both for the intrinsic production in terms of number of eggs, larvae, pupae, workers and their sum (*P* < 0.0001 in all cases) 60 days after the transplantation (Table 1). The average total intrinsic production in colonies without added pupae was 40.4 (±13.07 SD) individuals during the first 60 days of colony development. In comparison, the 100 pupae transplantation led to a 255% increase in the per capita queen production (143.6 brood individuals ± 8.87 SD), and the 300 pupae transplantation led to a 483% (235.8 brood individuals ± 12.94 SD) increase (Figure 1). Thus, the transplanted pupae stimulated the fertilized queen’s egg production and increased her production with approximately 2.6% and 1.6% per adopted pupa, respectively. The average total colony size (all intrinsic brood plus adopted workers) was 40.4 (±13.07 SD), 227.1 (±8.30 SD) and 442.7 (±15.70 SD), respectively, in the colonies that received 0, 100 and 300 pupae. In comparison to the treatment without pupae transplantation, the total number of individuals increased by 462% and 995%, respectively, in the 100 and 300 transplantation treatments (Figure 1).

**Table 1:** **Mean (± SD) number of intrinsic brood (eggs, larvae, pupae), imago workers and their total produced by the resident queen in the colonies 60 days after the transplantation of pupae**

Transplantation (no. of pupae)	Eggs per colony		Larvae per colony		Pupae per colony		Workers per colony		Total intrinsic production per colony	
	Mean (SD)	Two-way ANOVA	Mean (SD)	Two-way ANOVA	Mean (SD)	Two-way ANOVA	Mean (SD)	Two-way ANOVA	Mean (SD)	Two-way ANOVA
0	9.1 (5.43)	F_(2, 41)_ = 140.3	8.7 (5.10)	F_(2, 41)_ = 61.1	6.8 (3.64)	F_(2, 41)_ = 233.8	15.73 (5.22)	F_(2,41)_ = 1309.9	40.4 (13.07)	F_(2.41)_ = 1059.4
				*P* < 0.0001		*P* < 0.0001		*P* < 0.0001		*P* < 0.0001
		*P* < 0.0001								
100	19.8 (2.19)		19.9 (2.54)		16.4 (3.71)		87.4 (5.79)		143.6 (8.87)	
300	36.7 (5.09)		32.0 (8.09)		40.6 (5.45)		126.4 (7.42)		235.8 (12.94)	
Eggs before transplantation		F_(1, 41)_ = 0.1		F_(1,41)_ = 1.2		F_(1,41)_ = 0.0		F_(1,41)_ = 3.4		F_(1,41)_ = 2.5
										*P* = 0.11
								*P* = 0.07		
						*P* = 0.99				
				*P* = 0.26						
		*P* = 0.81								
Whole model		F_(3, 41)_ = 94.1		F_(3, 41)_ = 42.0		F_(3, 41)_ =156.4		F_(3, 41)_ = 882.3		F_(3, 41)_ =713.4
				*P* < 0.0001		*P* < 0.0001		*P* < 0.0001		*P* < 0.0001
		*P* < 0.0001

### Worker size

**Figure 2:**
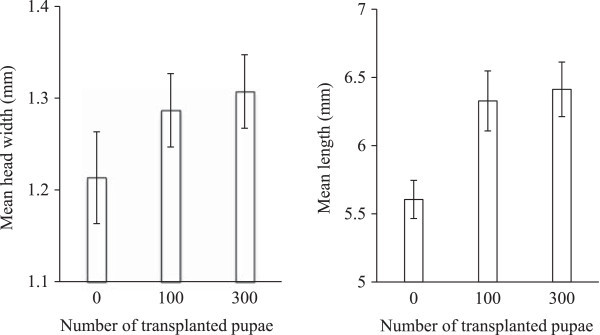
**The mean size (± SD) of nanitic worker pupae produced by the resident queen in the colonies 60 days after the transplantation of pupae.** Length and head width were significantly affected by transplantation rate (see Results).

The head width of the intrinsic hatching pupae showed that transplantation led to a significant increase in nanitic worker sizes (ANOVA including egg numbers before transplantation as a co-factor: F_(2, 116)_ = 50.23; *P* < 0.0001). A significant effect was also detected if pseudoreplication (Table 2) was avoided by using the average worker size of each colony as the unit for analysis (ANOVA including egg numbers before transplantation as a co-factor: F_(2, 8)_ = 6.1; *P* = 0.025). The average head width size was 1.21 (±0.05 SD), 1.29 (±0.04 SD) and 1.31 mm (±0.04 SD) in the colonies that received 0, 100 and 300 pupae, respectively (Figure 2). Thus, the transplanted pupae increased the head width of nanitic workers by 6.6% and 8.2%, respectively. Also nanitic worker length increased significantly with the number of transplanted pupae (ANOVA including egg numbers before transplantation as a co-factor: F_(2,116)_ = 212.70, *P* < 0.0001). The same was true if colony average sizes were used (ANOVA including egg numbers before transplantation as a co-factor: F_(2, 8)_ = 20.3; *P* = 0.0007) (Table 2). Mean (± SD) nanitic worker length was 5.61 (±0.14), 6.33 (±0.22) and 6.41 mm (±0.20), respectively, resulting in a 12.6% and a 14.5% increase for the 100 and 300 pupae transplantation, respectively (Figure 2). Thus, transplantation of pupae led not only to increased number of workers in colonies but also to larger individuals. Post hoc tests showed no significant differences in worker sizes (head width and worker length) between the 100 and 300 pupae treatments. In none of the above analyses did initial numbers of eggs show any significant effect.

**Table 2:** **ANOVAs comparing mean sizes of nanitic workers produced by the resident queen by transplantation treatment**

Transplantation (no. of pupae)	Length (mm)		Head width (mm)	
	Mean ± SD	Two-way ANOVA	Mean ± SD	Two-way ANOVA
0	5.61 ± 0.14	F_(2, 8)_ = 20.3	1.21 ± 0.05	F_(2,8)_ = 6.1
		*P =* 0.0007		*P =* 0.025
100	6.33 ± 0.22		1.29 ± 0.04	
300	6.41 ± 0.20		1.31 ± 0.04	
Eggs before transplantation		F_(1, 8)_ = 0.002		F_(1, 8)_ = 2.1
				*P =* 0.19
		*P =* 0.97		
Whole model		F_(3, 8)_ = 24.2		F_(3, 8)_ = 13.0
		*P =* 0.0002		*P =* 0.0019

## Discussion

### Pupae transplantation limit

An overall average of 76% of the transplanted pupae emerged to the adult stage and the resulting workers showed peaceful behavior towards the queens without signs of fights (based on casual observations), suggesting that non-nestmate pupae were readily accepted by the resident queens. However, as there was lower survival in the 300 pupae (69%) compared to 100 pupae treatment (83%), it seems the queens were unable to take care of all the pupae in the 300 pupae treatment and therefore pupae seem to require at least some amount of nursing (e.g. grooming, protection and temperature/humidity control by carrying pupae to optimal micro climates). This interpretation is based on the behavior shown by the queens in the 300 pupae treatments that culled some of the transplanted pupae, by throwing them out of the nesting space and into the water and since previous experience shows that unattended *O. longinoda* pupae rarely survive until hatching (mortality > 90%, I. Ouagoussounon, unpublished data). Culling makes sense if some nursing is required and if the queens were able to predict that their nursing abilities would be exceeded when faced with 300 pupae. It seemed, however, that the culling rate shown by the queens were not adequate as also the survival of the retained pupae was lower than in the 100 pupae treatment. Future studies separating causes of mortality and studies including a control treatment where pupae are alone without a queen to nurse them is required to gain more insight into the causes and mechanisms behind pupae mortality. Due to its applied character this was not addressed in the present study. Earlier studies have shown no difference in pupae proportions surviving between the number of pupae transplanted (of 30 and 60 pupae) per colony in *O. smaragdina* (Offenberg et al. 2012b; Peng et al. 2013) and between 50 and 100 pupae per queen in *O. longinoda* (Ouagoussounon et al. 2013). It therefore seems that the maximum number of pupae that can be adopted per queen without an associated significant loss in survival is somewhere between 100 and 300 pupae. Further, the present study showed that transplanted pupae increased queens’ brood production by 2.6% and 1.6% per adopted pupa, respectively, in the 100 and 300 pupae treatments, whereas Ouagoussounon et al. (2013) showed that *O. longinoda* transplanted pupae increased brood production by approximately 1.4% and 1.9% per adopted pupa, when transplanting 50 and 100 non-nestmate pupae. Thus, the per capita effect of transplanted pupae on queen production is optimized somewhere between the number of pupae transplanted of 100 and 300. That pupae were accepted in the colonies is also in agreement with findings by Krag et al. (2010) where *O. smaragdina* larvae were accepted by queenless worker colonies, and in agreement with other ant species where colony specific chemical cues are known not to develop until after worker eclosure (Lenoir et al. 2001).

### Effect on colony size

The transplantation of non-nestmate pupae directly increased colony size in proportion to the number of pupae added (minus pupae mortality); however, on top of this the queens also increased their brood production, as observed in previous studies (Offenberg et al. 2012b; Peng et al. 2013; Ouagoussounon et al. 2013). The combined effect resulted in a more than 5-fold increase in total colony size over the 60-day period with the 100 pupae transplantation, but with a more than 10-fold increase when 300 pupae were transplanted (Figure 1). Thus, even though survival and the increase in queen production per added pupa decline at the higher transplantation rate, is still pays off in terms of total colony size. In conclusion, if access to non-nestmate pupae is not restricted, high transplantation rates at 300 pupae per queen can still be used to manage and boost the growth of weaver ant colonies and in this way optimize their commercial utilization. The increase in total colony size in the present study (5.6-fold when transplanting 100 non-nestmate pupae) was higher than in the previous study on *O. longinoda* where a 3.9-fold increase was found (Ouagoussounon et al. 2013). The higher increase was probably caused by a 10-day longer growth period in the present experiment.

The direct cause of increased production by queens in association with pupae transplantation still needs to be resolved. Production could be triggered by the presence of high numbers of pupae (or the resulting workers) in the environment and a linked anticipation of food intake for the queen in the near future, as workers can forage and feed her soon after hatching from the pupal stage. Alternatively, availability of food (without the presence of workers) may stimulate production. In the latter case, the feeding of queens straight after their mating flight could be used as a measure to increase colony growth. The study of Cassill and Vinson (2007) speaks against this, as they found that only the number of late stage larvae was responsible for queen fertility in *Solenopsis invicta*. However, different mechanisms may apply to different ant species (Offenberg et al. 2012b).

### Nanitic worker size

The transplantation of major workers increased the size of intrinsic newly hatching workers. Worker size increased considerably between no transplantation and 100 pupae transplantation and less (and non-significantly) when comparing the 100 and 300 pupae transplantations (Figure 2). As a result the size range of workers in colonies receiving transplanted pupae increased, not only as large sized major workers from mature colonies were added (as pupae) to the nanitic workers, but also because the mean size of nanitic workers increased. If colony performance increased due to an increased task repertoire based on worker size range, then the adoption of pupae not only benefited the colony in terms of colony size, but also via better task handling. Larger workers may also directly benefit the efficiency of weaver ants in their control of pest species, if the presence of larger workers widens the prey spectrum and the hunting success of the colony (Dejean 1991; Crozier et al. 2010). Production of larger workers does not merely have implication for applied myrmecology. It is well known that adoption of robbed brood takes place under natural conditions in some ant species (Bartz and Hölldobler 1982; Rissing and Pollock 1987; Gadau et al. 2003; Kronauer et al. 2003). If the robbed brood not only adds numbers to the robbing colony but also accelerate its development of larger intrinsic workers and if this leads to increased colony performance, then an additional advantage is gained via brood robbing. Thus, the production of larger workers associated with pupae adoption may help to drive the evolution of brood robbing.

It is clear that the adoption of non-nestmate pupae had positive effects on both colony size and the size of workers. However, it should be noted, that also costs may be associated to colonies consisting of non-relatives. E.g. Linksvayer (2008) found that colonies of *Temnothorax curvispinosus* showed significantly lower production when relatedness among colony members decreased, suggesting that closely related phenotypes interact better than non-relatives. On the other hand, if this is also the case with *O. longinoda,* then this effect was much smaller than the benefit of being boosted with foreing pupae, as the adoption of pupae led to a net increase in production, at least within the experimental period of 60 days. Future studies are needed to test if long term detrimental effects are linked to “patchwork” colonies.

## Conclusion

This study showed that queenright *O. longinoda* colonies accepted foreign brood and that the presence of transplanted pupae increased queen production, number of workers (colony size), the mean worker size, and its range and variation. Also, the results showed there is a maximum limit of pupae a queen can nurse, but nevertheless high transplantation rates can still increase colony size more than 10-fold within a 60-day period. Future studies should test how fast incipient *O. longinoda* colonies can develop to their mature stage via repeated pupae transplantations.
